# Privacy-preserving ADP for secure tracking control of AVRs against unreliable communication

**DOI:** 10.3389/fnbot.2025.1549414

**Published:** 2025-01-29

**Authors:** Kun Zhang, Kezhen Han, Zhijian Hu, Guoqiang Tan

**Affiliations:** ^1^School of Astronautics, Beihang University, Beijing, China; ^2^School of Electrical Engineering, University of Jinan, Jinan, China; ^3^LAAS-CNRS, University of Toulouse, CNRS, Toulouse, France; ^4^Department of Aeronautical and Automotive Engineering, Loughborough University, Loughborough, United Kingdom

**Keywords:** adaptive dynamic programming, encryption and decryption, tracking control, optimal control, autonomous vehicle

## Abstract

In this study, we developed an encrypted guaranteed-cost tracking control scheme for autonomous vehicles or robots (AVRs), by using the adaptive dynamic programming technique. To construct the tracking dynamics under unreliable communication, the AVR's motion is analyzed. To mitigate information leakage and unauthorized access in vehicular network systems, an encrypted guaranteed-cost policy iteration algorithm is developed, incorporating encryption and decryption schemes between the vehicle and the cloud based on the tracking dynamics. Building on a simplified single-network framework, the Hamilton-Jacobi-Bellman equation is approximately solved, avoiding the complexity of dual-network structures and reducing the computational costs. The input-constrained issue is successfully handled using a non-quadratic value function. Furthermore, the approximate optimal control is verified to stabilize the tracking system. A case study involving an AVR system validates the effectiveness and practicality of the proposed algorithm.

## 1 Introduction

Autonomous vehicles or robots (AVRs) have rapidly transformed from a futuristic concept to a tangible reality, driving significant advancements in automotive technology. The advancement of autonomous vehicle technology has increasingly focused on improving tracking control systems, which are crucial for effective vehicle guidance (Pan et al., 2023). However, a persistent issue is the unreliable communication between a local vehicle and a reference vehicle, leading to discrepancies in signal reception and affecting tracking precision. In addition to these developments, the emergence of connected vehicles (Li et al., 2019a; Liu et al., 2023b), which leverages cloud computing for data processing and optimization, presents both opportunities and challenges. These systems function as cyber–physical systems (He et al., 2014; Zhang et al., 2014; Mohan et al., 2020), integrating computational and physical processes to enhance real-time data exchange and improve overall traffic management (Jiang et al., 2022; Li et al., 2019b). However, during communication between the vehicle and the cloud, the network's homogeneous and civilian nature makes it, particularly, vulnerable to attacks. This vulnerability, especially in the absence of robust security protocols, exposes these systems to cyber threats, including eavesdropping.

To enhance the security of vehicular cyber-physical systems, researchers from various fields, such as communication, control systems, and information theory, have developed various strategies to address cyberattacks across different layers (Han et al., 2024; Deng and Wen, 2021; Liu et al., 2021, 2023a). Various types of attacks, including denial-of-service (DoS) attacks, false data injection (FDI) attacks, and replay attacks, have been extensively studied (Teixeira et al., 2012; Li et al., 2024; Hu et al., 2023). These types of attacks share the characteristic of being active strategies designed to disrupt system functionality or manipulate transmitted data. Although defense mechanisms have made progress in countering such threats, majority of the existing methods primarily concentrate on detecting and mitigating explicit attacks, often overlooking the fundamental challenge of ensuring communication security. In vehicular cybersecurity, one of the critical issues is the threat of eavesdropping attacks (Yang et al., 2020; Wu et al., 2022). Unlike the direct and active nature of DoS and FDI attacks, eavesdropping operates passively, enabling attackers to intercept sensitive information while remaining undetected. This makes it a significant long-term threat that can compromise communication confidentiality and can even enable more destructive attacks. Addressing this challenge requires advanced encryption and privacy-preserving techniques to ensure secure communication. Although these methods are effective, they do not ensure optimal control performance at minimal energy cost, as they do not incorporate the principles of optimal control.

Optimal tracking control has become a cornerstone of modern control theory, with adaptive dynamic programming (ADP) algorithms attracting considerable interest in recent years (Lu et al., 2020; Mu et al., 2017b). For non-linear optimal control problems, the principal challenge lies in solving the Hamilton-Jacobi-Bellman (HJB) equation—a problem that is nearly intractable through exact mathematical methods. ADP techniques have offered a promising alternative by leveraging neural networks (NNs) to approximate optimal solutions, leading to significant advancements across fields such as automatic control and artificial intelligence (Mu et al., 2017a; Guo et al., 2024). For example, El-Sousy et al. (2021) designed a three-network structure to approximate the solution of the HJB equation for permanent-magnet synchronous motor servo drives. Wang et al. (2020) proposed an dual-network to approximate local Q-functions and control policies, solving optimal consensus control for non-linear multiagent systems. Furthermore, ADP-based optimal tracking control has been widely investigated (Dong et al., 2022; Song et al., 2023), including efforts to address input-constrained systems (Yang et al., 2023; Zhang et al., 2018). However, conventional ADP approaches, particularly those employing actor-critic frameworks, are frequently hindered by significant approximation errors introduced during iterative processes and NN training, thereby restricting their practical applicability.

To address these challenges, researchers have proposed several single-network ADP methodologies designed to streamline system architectures and enhance computational efficiency in handling nonlinear systems (Xu et al., 2023; Chen et al., 2021; Zou and Zhang, 2023). Chen et al. (2021) developed an event-triggered optimal control scheme for a macro–micro stage system, using a single critic NN to solve the modified HJB equation. In Guo et al. (2024), a distributed control strategy for attitude-constrained quadrotor unmanned aerial vehicle is proposed based on a critic network. Among the core ADP algorithms, value iteration and policy iteration (PI) have been widely employed, demonstrating robust performance in numerous applications (Zhang et al., 2020; Lin et al., 2023). However, the two-stage iterative procedures inherent in these methods frequently involve information transmission, which makes them susceptible to interception by adversaries. This vulnerability necessitates additional security measures, thereby increasing computational complexity and further constraining their applicability to complex systems. Although efforts to streamline computational burdens by eliminating actor networks have yielded progress, current ADP methods still inadequately address essential issues such as input saturation and ensuring reliable system performance, leaving these critical areas as potential opportunities for future research.

Unlike the previous studies, this article proposes an encrypted guaranteed-cost tracking control scheme for input-constrained tracking system with unreliable communication, and the main contributions are summarized as follows:

1. This article introduces an encrypted guaranteed-cost tracking control scheme for AVRs under unreliable communication. Compared with existing works, this is the first attempt to integrate ADP with encryption techniques, addressing both control performance and information security challenges in vehicular networks.
2. The designed privacy-preserving control method introduces a strategy to address eavesdropping attacks in control systems. By applying consistent output masking and encryption mechanisms at both the vehicle side and the cloud side, sensitive data and critical control information are effectively protected from potential breaches. This integrated approach ensures secure data transmission while maintaining the integrity and privacy of the control system.
3. A single-network structure with enhanced computational efficiency is proposed to approximate the HJB equation. Compared to conventional dual-network designs, the single-network structure reduces computational complexity while maintaining theoretical guarantees on weight error convergence and system stability. Additionally, input saturation is explicitly addressed through the adoption of a nonlinear value function, further enhancing the robustness.

## 2 Preliminaries and problem formulation

Consider an AVR operating in the X-Y plane, the position and orientation of the vehicle's mass center are represented by a posture vector


$$\dot{Z}:=\left[\begin{array}{c} x\left(t\right)\\ y\left(t\right)\\ \vartheta \left(t\right) \end{array}\right],$$

where *x*(*t*) and *y*(*t*) denote the horizontal and vertical positions, respectively, and ϑ(*t*) denotes the heading direction measured counterclockwise from the X-axis. The vehicle's motion is governed by the following kinematic model:


(1) $$\begin{array}{l} \dot{Z}=Ku\left(t\right)=\left[\begin{array}{cc} \cos\left(\vartheta \left(t\right)\right) & Y\sin\left(\vartheta \left(t\right)\right)\\ \sin\left(\vartheta \left(t\right)\right) & -Y\cos\left(\vartheta \left(t\right)\right)\\ 0 & 1 \end{array}\right]\left[\begin{array}{c} v\left(t\right)\\ w\left(t\right) \end{array}\right]. \end{array}$$

Here, *v*(*t*) and *w*(*t*) represent the vehicle's linear and rotational velocities, respectively, while Y is the distance between the vehicle's mass center and its drive axle; and K is the Jacobian matrix that links the control inputs to the vehicle's motion. So far, the control objectives are summarized in the following.

**Figure 1:**
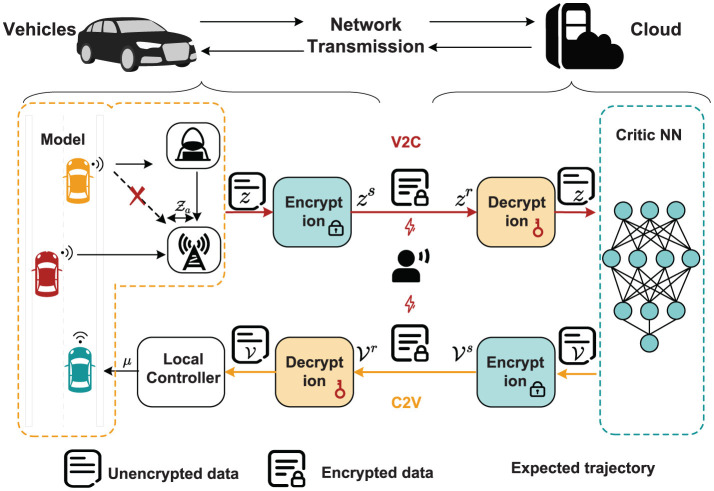
Proposed scheme for tracking process of AVRs.

*Control objective:* For an AVR under unreliable communication, design an ADP-based robust optimal controller with secure information exchange to drive the vehicle along the target, such that the following objectives are achieved:

1) *Robust tracking control objective:* For an AVR, Z_*c*_: = [*x*(*t*);*y*(*t*);ϑ(*t*)] to track the desired orbit Z_*d*_: = [*x*_*d*_(*t*);*y*_*d*_(*t*);ϑ_*d*_(*t*)] under malicious cyberattacks on the tracking process, as shown in Figure 1. Due to the occurrence of an attack, a small deviation arises between the received signal and the actual signal. This deviation, caused by malicious attacks, is defined as Z_*a*_: = [*x*_*a*_(*t*);*y*_*a*_(*t*);ϑ_*a*_(*t*)]. We assume that Z_*a*_ and its derivative are bounded.

With the minor difference Z_*a*_ caused by unreliable communication, following the framework in Zhang et al. (2022), we derive the tracking error system as


(2) $$\begin{array}{l} {\dot{Z}}_{e}=\left[\begin{array}{c} \cos\left(\vartheta_{e}\left(t\right)\right)v_{d}\left(t\right)+y_{e}\left(t\right)w_{d}\left(t\right)-v_{c}\left(t\right)+\gamma_{x}\left(t\right)\\ \sin\left(\vartheta_{e}\left(t\right)\right)v_{d}\left(t\right)-x_{e}\left(t\right)w_{d}\left(t\right)-w_{c}\left(t\right)+\gamma_{y}\left(t\right)\\ w_{d}\left(t\right)-w_{c}\left(t\right)+\gamma_{\vartheta }\left(t\right) \end{array}\right], \end{array}$$

where Z_*e*_: = [*x*_*e*_(*t*);*y*_*e*_(*t*);ϑ_*e*_(*t*)] denotes the tracking error posture, *v*_*d*_(*t*) and *w*_*d*_(*t*) are the desired linear and rotational velocities, *v*_*c*_(*t*) and *w*_*c*_(*t*) are the control inputs of the vehicle, and [γ_*x*_(*t*);γ_*y*_(*t*);γ_ϑ_(*t*)] captures the effect of cyberattacks on the received signals and given by


$$\left[\begin{array}{c} \gamma_{x}\left(t\right)\\ \gamma_{y}\left(t\right)\\ \gamma_{\vartheta }\left(t\right) \end{array}\right]=\left[\begin{array}{c} \cos\left(\vartheta_{c}\left(t\right)\right){ẋ}_{a}+\sin\left(\vartheta_{c}\left(t\right)\right){ẏ}_{a}\\ -\sin\left(\vartheta_{c}\left(t\right)\right){ẋ}_{a}+\cos\left(\vartheta_{c}\left(t\right)\right){ẏ}_{a}\\ {\dot{\vartheta }}_{a} \end{array}\right].$$

This model describes the dynamic behavior of the tracking error in AVR control.

To facilitate system description and control implementation, let us consider that *z* = [*x*_*e*_(*t*);*y*_*e*_(*t*);ϑ_*e*_(*t*)], *f*(*z*) = [cos(ϑ_*e*_(*t*))*v*_*d*_(*t*); sin(ϑ_*e*_(*t*))*v*_*d*_(*t*);*w*_*d*_(*t*)], *g*(*z*) = [−1, *y*_*e*_(*t*);0, L−*x*_*e*_(*t*);0, 1], and *u* = [*v*_*c*_(*t*), *w*_*c*_(*t*)]. The system (Equation 2) is rewritten as


(3) $$\begin{array}{l} ż=f\left(z\right)+g\left(z\right)u+\gamma , \end{array}$$

where *u* is control input and satisfies the asymmetric constrained set 𝔒 = {*u*||*u*| ≤ ℏ}. To follow the conventional optimal tracking architecture, we can rewrite the reference trajectory as follows


(4) $$\begin{array}{l} {ż}_{d}=f_{d}\left(z_{d}\right)+g_{d}\left(z_{d}\right)u_{d}, \end{array}$$

where *u*_*d*_ is the steady-state control input taking the following form


(5) $$\begin{array}{l} u_{d}=g_{d}^{-1}\left(z_{d}\right)\left({ż}_{d}-f_{d}\left(z_{d}\right)\right), \end{array}$$

where $g_{d}^{-1}\left(z_{d}\right)g_{d}\left(z_{d}\right)=I_{n}$, *I*_*n*_ denotes an *n* × *n* identify matrix.

Assumption 1. The unreliable communication γ(*t*) is bounded by $\bar{\gamma }$, that is $\|\bar{\gamma }\left(t\right)\|\leq \bar{\gamma }$, where $\bar{\gamma }$ is positive constant.

2) *Prevent eavesdropping objective:* As shown in Figure 1, the cloud handles monitoring, scheduling, optimization, and computation tasks, while the local controller is responsible for distributing control signals, albeit with limited data storage and processing capabilities. The cyberattack considered here is eavesdropping, where unauthorized interception of data during transmission allows attackers to steal sensitive system information, such as real-time control signals and operational states. To mitigate these risks, encryption and decryption mechanisms are implemented to safeguard the confidentiality and integrity of the transmitted data, ensuring secure communication and enhancing the system's overall reliability.

3) *Optimal control objective:* Based on the optimal control strategy, the AVR can achieve a compromise between performance and cost when running along a target, such that


(6) $$\begin{array}{ll} \min & \;J\left(z\right)=\int_{t}^{\infty }\gamma_{1}{\bar{\gamma }}^{2}+T\left(z,u\right)\text{d}s,\\ \text{s.t.} & \;ż=f\left(z\right)+g\left(z\right)u,u\in 𝔒, \end{array}$$

where $T\left(z,u\right)=z^{T}Qz+\bar{U}\left(\mu \right)$, which is the utility function with feedback control μ = *u* − *u*_*d*_, γ_1_ is positive constant, Q = Q^*T*^ > 0, and $\bar{U}\left(\cdot \right)$ is a positive definite non-quadratic integrand function.

## 3 Iterative algorithm design

In this section, based on the preceding analysis, the tracking problem is reformulated into a stabilization problem for the error dynamics. To address this, a cryptography-based controller is designed, which not only mitigates the impact of unreliable communication but also ensures the security of information transmission against eavesdropping.

### 3.1 Encryption and decryption algorithm design

To effectively counter eavesdropping attacks on data transmitted between the vehicle side and the cloud side, privacy-preserving rules are designed for both sides. The encryption and decryption formulas (Han et al., 2024) for each iteration are provided in the following.

1) *AVR to Cloud:*

*Encryption process:* At the vehicle side, the data *z* to be sent are extracted from Equation 3 and encrypted using Equation 7, resulting in the encrypted data *z*^*r*^. This encrypted data are then transmitted to the cloud. The encryption formula is as follows:


$$\{\begin{array}{ll} z^{s}=a(t)z+A\xi (t), & \left(7\text{a}\right)\\ a(t)=e^{\left(\delta_{1}\sum {\left\|V^{r}(z)(t-1)\right\|}_{2}^{2}\right)}, & \left(7\text{b}\right)\\ \xi (t)=\rho_{1}e^{-\left(t\mathrm{mod}\rho_{2}\right)}, & \left(7\text{c}\right) \end{array}$$

where *a*(*t*) and ξ(*t*) are encryption operators, δ_1_, ρ_1_, and ρ_2_ are constants, and *A* is the channel assignment matrices. To simplify the presentation of the method, it is assumed that V^*s*^(*z*)(*t* − 1) is already stored in the cloud. The value V(*z*) needs to be calculated on the cloud side. Its design is detailed in Section 3.2 and it serves as an essential component of the controller μ.

*Decryption process:* The cloud side receives the encrypted data *z*^*r*^ and decrypts it to recover the original data *z*. The decryption formula is as follows:


$$\{\begin{array}{ll} z=\frac{z^{r}-A\xi (t)}{c(t)}, & \left(8\text{a}\right)\\ c(t)=e^{\left(\delta_{1}\sum {\left\|V^{s}(z)(t-1)\right\|}_{2}^{2}\right)}, & \left(8\text{b}\right) \end{array}$$

where *c*(*t*) is the counterpart of *a*(*t*). It is observed that the design forms of the encryption operators *a*(*t*) and ξ(*t*), and encrypted expressions are shared between the vehicle side and the cloud. Furthermore, the parameters *A*, δ_1_, ρ_1_, and ρ_2_ are also shared.

2) *Cloud to AVR:*

*Encryption process:* After policy evaluation, the computed V(*z*) is encrypted using Equation 9 and sent back to the vehicle.


$$\{\begin{array}{ll} V^{s}(z)=b(t)V(z)+B\zeta (t), & \left(9\text{a}\right)\\ b(t)=e^{\left(\delta_{2}\sum {\left\|z^{r}\right\|}_{2}^{2}\right)}, & \left(9\text{b}\right)\\ \zeta (t)=\varrho_{1}e^{-\left(t\mathrm{mod}\varrho_{2}\right)}, & \left(9\text{c}\right) \end{array}$$

where *b*(*t*) and ζ(*t*) are encryption operators, δ_2_, ϱ_1_, and ϱ_2_ are constants, and *B* is the channel assignment matrices.

*Decryption process:* At the vehicle side, the received encrypted data V^*r*^(*z*) is decrypted using Equation 10 to recover V(*z*) for policy improvement.


$$\{\begin{array}{ll} V(z)=\frac{V^{r}(z)-B\zeta (t)}{d(t)},\; & \left(10\text{a}\right)\\ d(t)=e^{\left(\delta_{2}\sum {\left\|z^{s}\right\|}_{2}^{2}\right)}, & \left(10\text{b}\right) \end{array}$$

where *d*(*t*) is the counterpart of *b*(*t*). Similarly, the design forms of the encryption operators *b*(*t*) and ζ(*t*), and encrypted expressions are shared between the vehicle side and the cloud. Furthermore, the parameters *B*, δ_2_, ϱ_1_, and ϱ_2_ are also shared. At this point, a complete iteration of privacy-preserving processing has been completed.

From the above encryption and decryption processes, it can be observed that the introduced masking signals ξ(*t*) and ζ(*t*) and the encryption formula designs effectively ensure privacy during data transmission between the vehicle and the cloud. Notably, the data transmitted over the network are not the raw values *z* and V(*z*) but their encrypted counterparts, *z*^*s*^, *z*^*r*^, V^*s*^(*z*), and V^*r*^(*z*), which effectively prevent unauthorized entities from intercepting sensitive information.

### 3.2 Encrypted iterative algorithm design

The objective is to stabilize Equation 3 by constructing an encrypted iterative algorithm so that minimizing the performance index function, thereby reducing control costs and enhancing system security. Recalling Equation 6, the performance index is


(11) $$\begin{array}{l} V\left(z\right)=\int_{t}^{\infty }\left(\gamma_{1}{\bar{\gamma }}^{2}+z^{T}Qz+\bar{U}\left(\mu \right)\right)\text{d}s, \end{array}$$

where


(12) $$\begin{array}{l} \bar{U}\left(\mu \right)=\overset{m}{\underset{i=1}{\sum }}2\theta_{1}\int_{0}^{u_{i}-u_{d}}h^{-1}\left(\frac{\iota }{\theta_{1}}\right)r_{i}d\iota_{i}\\ =2\theta_{1}\int_{0}^{u-u_{d}}h^{-1}\left(\frac{\iota }{\theta_{1}}\right)Rd\iota , \end{array}$$

where R = diag{[*r*_1_, ..., *r*_*m*_]} > 0, $\iota ={\left[\iota_{1},...,\iota_{m}\right]}^{T}$. The function *h*(·) is assumed to be a monotonic odd function satisfying *h*(0) = 0. For the purposes of this article, *h*(·) is specifically selected as *h*(*x*) = (*e*^*z*^ − *e*^−*z*^)/(*e*^*z*^ + *e*^−*z*^).

According to the optimal control theory, Equation 11 is a Lyapunov function for the Equation 3 and the Hamiltonian function can be derived as


(13) $$\begin{array}{l} H\left(z,\mu ,V\left(z\right)\right)=\gamma_{1}{\bar{\gamma }}^{2}+z^{T}Qz+\bar{U}\left(\mu \right)+\nabla V\left(z\right)(f\left(z\right)+g\left(z\right)u+\gamma ), \end{array}$$

with $\nabla V\left(z\right)=\frac{\partial V\left(z\right)}{\partial z}$. On defining V^*^(*z*) as the minimum value of Equation 11, based on Bellman's principle of optimality, we have


(14) $$\begin{array}{l} 0=H\left(z,\mu ,V^{*}\left(z\right)\right)\\ =\gamma_{1}{\bar{\gamma }}^{2}+z^{T}Qz+\bar{U}\left(\mu \right)+\nabla V^{*}\left(z\right)(f\left(z\right)+g\left(z\right)u^{*}+\gamma ), \end{array}$$

and the optimal control u^*^ is obtained from $\frac{\partial H\left(z,\mu ,V^{*}\left(z\right)\right)}{\partial u^{*}}=0$:


(15) $$\begin{array}{l} u^{*}=\theta_{1}\tanh(-\frac{1}{2\theta_{1}}R^{-1}g^{T}\left(z\right)\nabla V^{*}\left(z\right))+u_{d}. \end{array}$$

Substituting Equation 15 into Equation 12 yields


(16) $$\begin{array}{l} \bar{U}\left(\mu^{*}\right)=\nabla V^{*T}\left(z\right)g\left(z\right)\tanh\left(D\left(z\right)\right)+\theta_{1}^{2}\overset{m}{\underset{i=1}{\sum }}\ln\left(1-\tanh^{2}\left(D_{i}\left(z\right)\right)\right), \end{array}$$

where $D\left(z\right)=\frac{1}{2\theta_{1}}R^{-1}g^{T}\left(z\right)\nabla V^{*}\left(z\right)$ and $\mu^{*}=u^{*}-u_{d}$. Then, the HJB equation can be derived as


(17) $$\begin{array}{l} H\left(z,\mu^{*},V^{*}\left(z\right)\right)=\gamma_{1}{\bar{\gamma }}^{2}+z^{T}Qz+\nabla V^{*}\left(z\right)(f\left(z\right)+\gamma )\\ +\theta_{1}^{2}\overset{m}{\underset{i=1}{\sum }}\ln\left(1-\tanh^{2}\left(D_{i}\left(z\right)\right)\right)=0. \end{array}$$

As highlighted in the preceding analysis, obtaining the optimal controller in Equation 15 necessitates solving the HJB Equation 17, a task well-known for its considerable computational and analytical challenges. To overcome this challenge, an iterative algorithm based on ADP is employed to obtain an approximate solution. The details of this iterative algorithm are presented in Algorithm 1.

**Algorithm 1:**
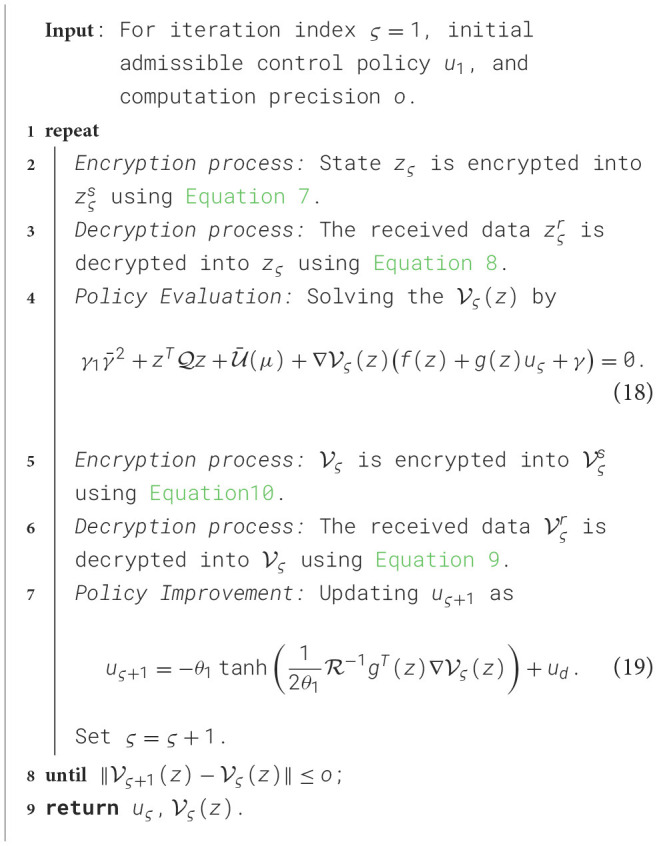
Encrypted guaranteed cost policy iteration algorithm.

Lemma 1. By utilizing the encrypted PI process as described in Algorithm 1, which incorporates encryption and decryption steps for secure control of the tracking error dynamics in an AVR, the resulting control *u*_ς_ ensures the asymptotic stability of the system dynamics. Additionally, V_ς_(*z*) will converge to the optimal value function V^*^(*z*) as ς → ∞, ensuring that *u*_ς_ converges to the optimal control u^*^.

*Proof*. Initially, without iterations, the control *u*_1_ is considered admissible. For ∀*u*_ς_ produced during iterations, consider the Lyapunov function V_ς_(*z*), which satisfies


(20) $$\begin{array}{l} {\dot{V}}_{\varsigma }\left(z\right)=\nabla V_{\varsigma }\left(z\right)ż\\ =\nabla V_{\varsigma }\left(z\right)(f\left(z\right)+g\left(z\right)u_{\varsigma }+\gamma ). \end{array}$$

According to HJB Equation 17, we can drive


(21) $$\begin{array}{l} \nabla V_{\varsigma }\left(z\right)(f\left(z\right)+g\left(z\right)u_{\varsigma }+\gamma )=-\gamma_{1}{\bar{\gamma }}^{2}-z^{T}Qz-\bar{U}\left(\mu_{\varsigma }\right), \end{array}$$

where μ_ς_ = *u*_ς_ − *u*_*d*_. Then, substituting Equation 21 into Equation 22 yields


(22) $$\begin{array}{l} {\dot{V}}_{\varsigma }\left(z\right)=-\gamma_{1}{\bar{\gamma }}^{2}-z^{T}Qz-\bar{U}\left(\mu_{\varsigma }\right)\leq 0. \end{array}$$

Therefore, the iteration process ensures that the error dynamics remain asymptotically stable. Moreover, policy improvement is achieved by minimizing the associated value function, consistent with the Kleinman method, guaranteeing convergence. As the iteration count ς → ∞, $V_{\varsigma }\left(z\right)\to V^{*}\left(z\right)$, and $u_{\varsigma }\to u^{*}$ hold. This concludes the proof.     □

Based on Lemma 1, the iterative process, enhanced with secure encryption and decryption, converges, leading to optimal control as the approximation errors diminish.

## 4 Critic neural network design

In this section, this study employs the fundamental update equations of PI to design a NN, utilizing the critic neural network (CNN) to approximate the solution of the HJB Equation 17 during each iteration step. Therefore, based on the universal approximation property of NNs, there exist ideal weights W^*^ such that the ideal value function can be approximated as


(23) $$\begin{array}{l} V^{*}\left(z\right)=W^{*T}\varphi \left(z\right)+\epsilon_{1}\left(z\right), \end{array}$$

where φ(*z*) ∈ ℝ^α^ denotes activation functions and α is the number of neurons. Utilizing Equation 23, HJB Equation 17 becomes


(24) $$\begin{array}{l} \gamma_{1}{\bar{\gamma }}^{2}+z^{T}Qz+\left(W^{*T}\nabla \varphi \left(z\right)+\nabla \epsilon_{1}^{T}\left(z\right)\right)(f\left(z\right)+\gamma )\\ +\theta_{1}^{2}\overset{m}{\underset{i=1}{\sum }}\ln\left(1-\tanh^{2}\left(H_{i}\left(z\right)\right)\right)=0, \end{array}$$

where


(25) $$\begin{array}{l} H_{i}\left(z\right)=H_{1i}\left(z\right)+H_{2i}\left(z\right)\\ =\frac{1}{2\theta }R^{-1}g^{T}\left(z\right)\nabla \varphi^{T}\left(z\right)W^{*}+\frac{1}{2\theta_{1}}R^{-1}g^{T}\left(z\right)\nabla \epsilon_{1}^{T}\left(z\right), \end{array}$$

with $\nabla \varphi \left(z\right)=\frac{\partial \varphi_{1}}{\partial z}$ and $\nabla \epsilon_{1}\left(z\right)=\frac{\partial \varphi }{\partial z}$. Therefore, by defining residual error ϵ_*H*_, Equation 24 can be rewritten as


(26) $$\begin{array}{l} \gamma_{1}{\bar{\gamma }}^{2}+z^{T}Qz+W^{*T}\nabla \varphi \left(z\right)(f\left(z\right)+\gamma )+\epsilon_{H}\\ +\theta_{1}^{2}\overset{m}{\underset{i=1}{\sum }}\ln\left(1-\tanh^{2}\left(H_{1i}\left(z\right)\right)\right)=0, \end{array}$$

where


(27) $$\begin{array}{l} \epsilon_{H}=\nabla \epsilon_{1}^{T}\left(z\right)(f\left(z\right)+\gamma )-\theta_{1}^{2}\overset{m}{\underset{i=1}{\sum }}\frac{1}{O_{1i}\left(z\right)}\tanh\left(O_{2i}\left(z\right)\right)\\ \left(1-\tanh^{2}\left(O_{2i}\left(z\right)\right)\right), \end{array}$$

with $O_{1i}\left(z\right)\in \left[1-\tanh^{2}\left(D_{i}\left(z\right)\right),1-\tanh^{2}\left(H_{1i}\left(z\right)\right)\right]$, O_2*i*_(*z*) ∈ [D_*i*_(*z*), H_1*i*_(*z*)]. Note that if the number of hidden layer neurons α is sufficiently large, the residual error ϵ_*H*_ will approach zero. Based on the Lipschitz assumption of the system dynamics, this ϵ_*H*_ is bounded within a compact set, that is, $\|\epsilon_{H}\|\leq {\bar{\epsilon }}_{H}$. Therefore, based on Equation 23 the ideal optimal control is


(28) $$\begin{array}{l} u^{*}=\theta_{1}\tanh(-\frac{1}{2\theta_{1}}R^{-1}g^{T}\left(z\right)\nabla \varphi^{T}W^{*})+u_{d}+\epsilon_{2} \end{array}$$

where $\epsilon_{2}=-\frac{1}{2}\overset{m}{\underset{i=1}{\sum }}\left(1-\tanh^{2}\left(\psi_{i}\right)\right)R^{-1}g^{T}\left(z\right)\nabla \epsilon_{1}$, ψ_*i*_ ∈ [D_*i*_, H_1*i*_].

Since the ideal weight is unknown, the approximated value function is


(29) $$\begin{array}{l} \hat{V}\left(z\right)={\hat{W}}^{T}\varphi \left(z\right), \end{array}$$

where $\hat{W}$ is approximated value of W^*^. Then, we can get


(30) $$\begin{array}{l} û=-\theta_{1}\tanh(\frac{1}{2\theta_{1}}R^{-1}g^{T}\left(z\right)\nabla \varphi^{T}\left(z\right)\hat{W})+u_{d}. \end{array}$$

Thus, approximated Hamiltonian function is


(31) $$\begin{array}{l} H\left(z,\hat{\mu },\hat{V}\left(z\right)\right)=\gamma_{1}{\bar{\gamma }}^{2}+z^{T}Qz+{\hat{W}}^{T}\nabla \varphi \left(z\right)(f\left(z\right)+\gamma )\\ +\theta_{1}^{2}\overset{m}{\underset{i=1}{\sum }}\ln\left(1-\tanh^{2}\left({\hat{H}}_{1i}\left(z\right)\right)\right)\\ :={\hat{\epsilon }}_{H}, \end{array}$$

where ${\hat{\epsilon }}_{H}$ is the residual error due to NN approximation error.

Furthermore, let us consider $E=\frac{1}{2}{\hat{\epsilon }}_{H}^{T}{\hat{\epsilon }}_{H}$, and to ensure that $\hat{W}$ converge toward the optimal weights W^*^, the weight update formula (Zhang et al., 2018) is


(32) $$\begin{array}{l} {\dot{\hat{W}}}_{1}=-\eta \frac{\tau }{\varpi^{2}}{\hat{\epsilon }}_{H}+\frac{\eta }{2}\kappa \nabla \varphi_{1}(g\left(I-M\left({\hat{H}}_{1}\right)\right)g^{T})\nabla V_{a}\\ +\eta (-\theta_{1}\nabla \varphi gS\frac{\tau^{T}}{\varpi }\hat{W}-\left(K_{2}-K_{1}\tau^{T}\right)\hat{W}), \end{array}$$

where η is learning rate, τ = ∇φ(*z*)(*f*(*z*)+*g*(*z*)û+γ), ϖ = τ^*T*^ τ + 1, and K_1_ and K_2_ are a tuning matrix. $M=\text{diag}\left\{\tanh^{2}\left({\hat{H}}_{1i}\right)\right\}$, $S=\text{sgn}\left({\hat{H}}_{1}\right)-\tanh\left({\hat{H}}_{1}\right)$. Based on the Lemma 2 by Zhang et al. (2018), V_*a*_ denotes Lyapunov function, and if ∇V_*a*_(*f*(*z*)+*gû* + γ) > 0, then κ = 0, else κ = 1. Defining $\tilde{W}=W^{*}-\hat{W}$, we obtain


(33) $$\begin{array}{l} \dot{\tilde{W}}=-\eta \tau \tau^{T}\tilde{W}+\eta \frac{\tau }{\varpi }\left(\theta_{1}{\tilde{W}}^{T}\nabla \varphi gS+{\overset{⌣}{\epsilon }}_{H}\right)\\ -\frac{\eta }{2}\kappa \nabla \varphi_{1}g(I-M\left({\hat{H}}_{1}\right))g^{T}\nabla V_{a}\\ +\eta \theta_{1}\nabla \varphi gS\frac{\tau^{T}}{\varpi }\hat{W}+\eta \left(K_{2}-K_{1}\tau^{T}\right)\hat{W}, \end{array}$$

with ${\overset{⌣}{\epsilon }}_{H}=\theta_{1}W^{T}\nabla \varphi g(\text{sgn}\left(H_{1}\right)-\text{sgn}\left({\hat{H}}_{1}\right))+2\theta_{1}^{2}\bar{H}-\epsilon_{H}$, $\bar{H}=\overset{m}{\underset{i=1}{\sum }}\ln\frac{1+\text{exp}\left(-2H_{1i}\right)}{1+\text{exp}\left(-2{\hat{H}}_{1i}\right)}$.

Theorem 1. For the optimal control policy described in Equation 30, the weight tuning law of the CNN is determined by the update formula provided in Equation 32. Under this design, the error dynamic system *z* and the weight errors $\tilde{W}$ are uniformly ultimately bounded (UUB).

*Proof*. Define the Lyapunov function as L = L_1_ + L_2_, where


(34) $$\begin{array}{l} L_{1}=\frac{1}{2}{\tilde{W}}^{T}\eta^{-1}\tilde{W},\;L_{2}=V_{a}\left(z\right). \end{array}$$

First, along Equation 33, the derivative of L_2_ is


(35) $$\begin{array}{l} {\dot{L}}_{1}={\tilde{W}}^{T}\eta^{-1}{\dot{\tilde{W}}}_{1}\\ ={\tilde{W}}^{T}\eta^{-1}\{-\eta \tau \tau^{T}\tilde{W}+\eta \frac{\tau }{\varpi }\left(\theta_{1}{\tilde{W}}^{T}\nabla \varphi gS+{\overset{⌣}{\epsilon }}_{H}\right)-\frac{\eta }{2}\kappa \nabla \varphi_{1}(g\left(I-M\left({\hat{H}}_{1}\right)\right)g^{T})\nabla V_{a}\\ +\eta \theta_{1}\nabla \varphi gS\frac{\tau^{T}}{\varpi }\hat{W}+\eta \left(K_{2}-K_{1}\tau^{T}\right)\hat{W}\}\\ =-{\tilde{W}}^{T}\tau \tau^{T}\tilde{W}+\theta_{1}{\tilde{W}}^{T}\nabla \varphi gS\frac{\tau^{T}}{\varpi }{\tilde{W}}^{T}+{\overset{⌣}{\epsilon }}_{H}\frac{\tau^{T}}{\varpi }{\tilde{W}}^{T}-\frac{1}{2}\kappa \nabla V_{a}^{T}g(I-M\left({\hat{H}}_{1}\right))g^{T}\nabla \varphi^{T}\tilde{W}\\ +\theta_{1}{\tilde{W}}^{T}\nabla \varphi gS\frac{\tau^{T}}{\varpi }W^{*}-\theta_{1}{\tilde{W}}^{T}\nabla \varphi gS\frac{\tau^{T}}{\varpi }\tilde{W}+{\tilde{W}}^{T}\left(K_{2}-K_{1}\tau^{T}\right)\hat{W}\\ =-{\tilde{W}}^{T}\tau \tau^{T}\tilde{W}+{\overset{⌣}{\epsilon }}_{H}\frac{\tau^{T}}{\varpi }{\tilde{W}}^{T}-\frac{1}{2}\kappa \nabla V_{a}^{T}g(I-M\left({\hat{H}}_{1}\right))g^{T}\nabla \varphi^{T}\tilde{W}\\ +\theta_{1}{\tilde{W}}^{T}\nabla \varphi gS\frac{\tau^{T}}{\varpi }W^{*}+{\tilde{W}}^{T}\left(K_{2}-K_{1}\tau^{T}\right)\hat{W}\\ =-P^{T}AP+P^{T}B-\frac{1}{2}\kappa \nabla V_{a}^{T}g(I-M\left({\hat{H}}_{1}\right))g^{T}\nabla \varphi^{T}\tilde{W}, \end{array}$$

where


$$\begin{array}{l} P=\left[\begin{array}{c} {\tilde{W}}^{T}\tau \\ {\tilde{W}}^{T} \end{array}\right],A=\left[\begin{array}{cc} I & -\frac{1}{2}K_{1}^{T}\\ -\frac{1}{2}K_{1} & K_{2} \end{array}\right], \end{array}$$

$$\begin{array}{l} B=\left[\begin{array}{c} -\frac{1}{\varpi }{\overset{⌣}{\epsilon }}_{H}\\ \left(\theta_{1}{\tilde{W}}^{T}\nabla \varphi gS\frac{\tau^{T}}{\varpi }+K_{2}-K_{1}\tau^{T}\right)W^{*} \end{array}\right]. \end{array}$$

Supposing that $\|W^{*}\|\leq {\bar{W}}_{1}$, ${\bar{W}}_{1}>0$, and due to ${\overset{⌣}{\epsilon }}_{H}$ is bound, $\|B\|\leq \bar{B}$, $\bar{B}>0$. Therefore, ${\dot{L}}_{1}$ is


(36) $$\begin{array}{ll} {\dot{L}}_{1}\leq & -\lambda_{\min}\left(A\right)\|P|{|}^{2}+\bar{B}\|P\|-\frac{1}{2}\kappa \nabla V_{a}^{T}g(I-M\left({\hat{H}}_{1}\right))g^{T}\nabla \varphi^{T}\tilde{W}. \end{array}$$

Owing to κ of L_2_, $\dot{L}$ is divided into two parts. For κ = 0, we have


(37) $$\begin{array}{ll} \dot{L}\leq & \nabla V_{a}^{T}ż-\lambda_{\min}\left(A\right)\|P|{|}^{2}+\bar{B}\|P\|. \end{array}$$

From a study by Rudin et al. (1964), we can know $\nabla V_{a}^{T}ż<-\|\nabla V_{a}\|z_{m}<0$, ||*z*|| ≤ *z*_*m*_, *z*_*m*_ > 0, thus, $\dot{L}$ becomes


$$\begin{array}{ll} \dot{L}\leq & -\|\nabla V_{a}\|z_{m}-\lambda_{\min}\left(A\right)(\|P\|-\frac{\bar{B}}{2\lambda_{\min}\left(A\right)}{)}^{2}+\frac{{\bar{B}}^{2}}{4\lambda_{\min}\left(A\right)}. \end{array}$$

Moreover, $\dot{L}<0$ if


(38) $$\begin{array}{ll} \|\nabla V_{a}\|> & \frac{{\bar{B}}^{2}}{4z_{m}\lambda_{\min}\left(A\right)}, \end{array}$$

or


(39) $$\begin{array}{l} \|P\|>\frac{\bar{B}}{2\lambda_{\min}\left(A\right)}. \end{array}$$

According to Equation 39, we can derive


(40) $$\begin{array}{l} \|\tilde{W}\|>\frac{2\bar{B}}{\sqrt{5}\lambda_{\min}\left(A\right)}. \end{array}$$

For κ = 1, $\dot{L}$ is


(41) $$\begin{array}{l} \dot{L}\leq \nabla V_{a}^{T}ż-\lambda_{\min}\left(A\right)\|P|{|}^{2}+\bar{B}\|P\|\\ -\frac{1}{2}\kappa \nabla V_{a}^{T}g(I-M\left({\hat{H}}_{1}\right))g^{T}\nabla \varphi^{T}\tilde{W}\\ \leq \nabla V_{a}^{T}\left(f+gû+\gamma \right)-\lambda_{\min}\left(A\right)\|P|{|}^{2}+\bar{B}\|P\|\\ -\frac{1}{2}\nabla V_{a}^{T}g(I-M\left({\hat{H}}_{1}\right))g^{T}\nabla \varphi^{T}\tilde{W}. \end{array}$$

Regarding $\tanh\left(H_{1}\right)-\tanh\left({\hat{H}}_{1}\right):=\overset{⌣}{H}$, using the Taylor series, we know


$$\begin{array}{l} \overset{⌣}{H}=\frac{1}{2\theta_{1}}(I-M\left({\hat{H}}_{1}\right))g^{T}\nabla \varphi^{T}\tilde{W}+o({\left(H_{1}-{\hat{H}}_{1}\right)}^{2}), \end{array}$$

where $o({\left(H_{1}-{\hat{H}}_{1}\right)}^{2})$ is the higher order term and satisfies


(42) $$\begin{array}{l} \left\|o({\left(H_{1}-{\hat{H}}_{1}\right)}^{2})\right\|\\ \leq \|\overset{⌣}{H}\|+\frac{1}{2\theta_{1}}(I-M\left({\hat{H}}_{1}\right))g^{T}\nabla \varphi^{T}\tilde{W}\\ \leq \|\tanh\left(H_{1}\right)\|+\|\tanh\left({\hat{H}}_{1}\right)\|+\frac{1}{2\theta_{1}}(I-M\left({\hat{H}}_{1}\right))g^{T}\nabla \varphi^{T}\tilde{W}\\ =(\sum_{i=1}^{m}|\tanh\left(H_{1}\right){|}^{2}{)}^{\frac{1}{2}}\\ +(\sum_{i=1}^{m}|\tanh\left({\hat{H}}_{1}\right){|}^{2}{)}^{\frac{1}{2}}+\frac{1}{2\theta_{1}}(I-M\left({\hat{H}}_{1}\right))g^{T}\nabla \varphi^{T}\tilde{W}\\ \leq 2\sqrt{m}+\frac{1}{\theta_{1}}ḡ\bar{\varphi }\|\tilde{W}\|, \end{array}$$

where ||*g*|| ≤ ḡ, ḡ > 0 and $\|\nabla \varphi \|\leq \bar{\varphi }$, $\bar{\varphi }>0$.

Recalling Equations 28–30, the term in Equation 41 with respect to ∇V_*a*_*g* can be written as


(43) $$\begin{array}{l} \nabla V_{a}^{T}(gû-\frac{1}{2}g(I-M\left({\hat{H}}_{1}\right))g^{T}\nabla \varphi^{T}\tilde{W})\\ =-\theta_{1}\nabla V_{a}^{T}g\tanh\left(H_{1}\right)+\theta_{1}\nabla V_{a}^{T}go({\left(H_{1}-{\hat{H}}_{1}\right)}^{2})\\ =\nabla V_{a}^{T}gu^{*}-\nabla V_{a}^{T}\epsilon_{2}+\theta_{1}\nabla V_{a}^{T}go({\left(H_{1}-{\hat{H}}_{1}\right)}^{2}). \end{array}$$

Until now, Equation 41 can be rewritten as


(44) $$\begin{array}{l} \dot{L}\leq \nabla V_{a}^{T}\left(f+gu^{*}+\gamma \right)-\lambda_{\min}\left(A\right)\|P|{|}^{2}+\bar{B}\|P\|-\nabla V_{a}^{T}\epsilon_{2}+\theta_{1}\nabla V_{a}^{T}go({\left(H_{1}-{\hat{H}}_{1}\right)}^{2})\\ \leq \|\nabla V_{a}\|\|f+gu^{*}\|+\|\nabla V_{a}\|\|\gamma \|-\lambda_{\min}\left(A\right)\|P|{|}^{2}+\bar{B}\|P\|-\nabla V_{a}^{T}\epsilon_{2}\\ +\theta_{1}\nabla V_{a}^{T}go({\left(H_{1}-{\hat{H}}_{1}\right)}^{2})\\ \leq -\lambda_{\min}\left(A\right)\|P|{|}^{2}+\bar{B}\|P\|-\lambda_{\min}\left(C\right)\|\nabla V_{a}|{|}^{2}+{\bar{\epsilon }}_{2}ḡ\|\nabla V_{a}\|+2\theta_{1}\sqrt{m}ḡ\|\nabla V_{a}\|\\ +\bar{\gamma }\|\nabla V_{a}\|+{ḡ}^{2}\bar{\varphi }\|\nabla V_{a}\|\|\tilde{W}\|\\ =-\lambda_{\min}\left(A\right)\|P|{|}^{2}+\bar{B}\|P\|-\lambda_{\min}\left(C\right)\|\nabla V_{a}|{|}^{2}+\omega \|\nabla V_{a}\|+{ḡ}^{2}\bar{\varphi }\|\nabla V_{a}\|\|\tilde{W}\|, \end{array}$$

where $\|\epsilon_{2}\|\leq {\bar{\epsilon }}_{2}$, ${\bar{\epsilon }}_{2}>0$. Let ℓ_1_ and ℓ_2_ satisfy 0 < ℓ_1_ < 1, 0 < ℓ_2_ < 1, and ℓ_1_ + ℓ_2_ = 1. Then, Equation 44 can be rewritten as


$$\begin{array}{l} \dot{L}\leq -\frac{4\ell_{2}\lambda_{\min}\left(C\right)\lambda_{\min}\left(A\right)-{ḡ}^{4}{\bar{\varphi }}^{2}}{4\ell_{2}\lambda_{\min}\left(C\right)}(\|P\|-\frac{2\ell_{2}\lambda_{\min}\left(C\right)\bar{B}}{4\ell_{2}\lambda_{\min}\left(C\right)\lambda_{\min}\left(A\right)-{ḡ}^{4}{\bar{\varphi }}^{2}}{)}^{2}\\ -\ell_{1}\lambda_{\min}\left(C\right)(\|\nabla V_{a}\|-\frac{\omega }{2\ell_{1}\lambda_{\min}\left(C\right)}{)}^{2}-\ell_{1}\lambda_{\min}\left(C\right)(\|\nabla V_{a}\|-\frac{{ḡ}^{2}\bar{\varphi }}{2\ell_{1}\lambda_{\min}\left(C\right)}\|\tilde{W}\|{)}^{2}\\ +\frac{\ell_{2}\lambda_{\min}\left(C\right){\bar{B}}^{2}}{4\ell_{2}\lambda_{\min}\left(C\right)\lambda_{\min}\left(A\right)-{ḡ}^{4}{\bar{\varphi }}^{2}}+\frac{\omega^{2}}{4\ell_{1}\lambda_{\min}\left(C\right)}\\ =-\frac{\omega_{1}}{4\ell_{2}\lambda_{\min}\left(C\right)}(\|P\|-\frac{2\ell_{2}\lambda_{\min}\left(C\right)\bar{B}}{\omega_{1}}{)}^{2}-\ell_{1}\lambda_{\min}\left(C\right)(\|\nabla V_{a}\|-\frac{\omega }{2\ell_{1}\lambda_{\min}\left(C\right)}{)}^{2}\\ -\ell_{1}\lambda_{\min}\left(C\right)(\|\nabla V_{a}\|-\frac{{ḡ}^{2}\bar{\varphi }}{2\ell_{1}\lambda_{\min}\left(C\right)}\|\tilde{W}\|{)}^{2}+\omega_{2}. \end{array}$$

Therefore, $\dot{L}<0$ if


(45) $$\begin{array}{ll} \|\nabla V_{a}\|> & \frac{{ḡ}^{2}\bar{\varphi }}{2\ell_{1}\lambda_{\min}\left(C\right)}+\sqrt{\frac{\omega_{1}}{\ell_{1}\lambda_{\min}\left(C\right)}}, \end{array}$$

or


(46) $$\begin{array}{ll} \|P\|> & \frac{2\ell_{2}\lambda_{\min}\left(C\right)\bar{B}}{\omega_{1}}+2\sqrt{\frac{\ell_{2}\lambda_{\min}\left(C\right)\omega_{2}}{\omega_{1}}}. \end{array}$$

Similar to Equation 40, we can derive


(47) $$\begin{array}{ll} \|\tilde{W}\|> & \frac{4\ell_{2}\lambda_{\min}\left(C\right)\bar{B}}{\sqrt{5}\omega_{1}}+4\sqrt{\frac{\ell_{2}\lambda_{\min}\left(C\right)\omega_{2}}{5\omega_{1}}}. \end{array}$$

By considering the two cases, κ = 0 and κ = 1, and based on the derived results as expressed in Equations 38–40 and Equations 45–47, we can conclude that the function ∇V_*a*_ and the error weights $\tilde{W}$ are UUB. Furthermore, knowing that V_*a*_ is in polynomial form, it follows that the error *z* is also UUB.

**Figure 2:**
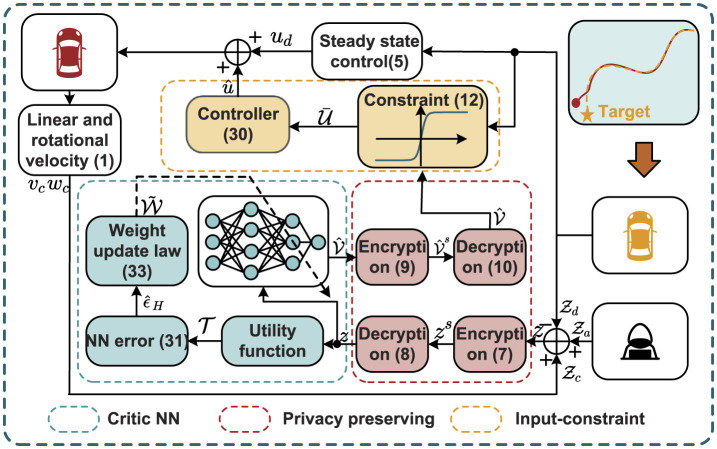
Illustration of tracking for AVRs subject to privacy protection.

Remark 1. The algorithm designed in this article is depicted in Figure 2, where Algorithm 1 is implemented using a CNN. The CNN generates the estimated value function $\hat{V}$, which is subsequently used to derive the approximated optimal control law û based on Equation 30. In contrast to the constrained optimal control designs presented in the studies by Zou and Zhang (2023); Chen et al. (2021), this work integrates privacy-preserving mechanisms during information transmission by leveraging encryption and decryption techniques. This incorporation not only safeguards data confidentiality but also enhances the overall security and reliability of the proposed algorithm.

## 5 Simulation results

To analyze the tracking performance of the AVR, we conduct simulations based on a predefined tracking error dynamic model. The tracking error dynamics ${\dot{Z}}_{e}$ is modeled as


(48) $$\begin{array}{l} \left[\begin{array}{c} {ẋ}_{e}\\ {ẏ}_{e}\\ {\dot{\vartheta }}_{e} \end{array}\right]=\left[\begin{array}{c} \cos\left(\vartheta_{e}\right)v_{d}\\ \sin\left(\vartheta_{e}\right)v_{d}\\ w_{d} \end{array}\right]+\left[\begin{array}{cc} -1 & y_{e}\\ 0 & Y-x_{e}\\ 0 & -1 \end{array}\right]u+\gamma , \end{array}$$

where Y represents the distance from the vehicle's center of mass to the rear axle, set to Y = −1.2m in this article. The desired reference trajectory is initialized with the state:


$${\left[x_{d}\left(0\right),y_{d}\left(0\right),\vartheta_{d}\left(0\right)\right]}^{T}={\left[0,0,0\right]}^{T},$$

and the vehicle's trajectory is initialized with the state:


$${\left[x_{c}\left(0\right),y_{c}\left(0\right),\vartheta_{c}\left(0\right)\right]}^{T}={\left[-2.5,2.5,-0.5\right]}^{T}.$$

Consequently, the initial value of error denotes


$${\left[x_{e}\left(0\right),y_{e}\left(0\right),\vartheta_{e}\left(0\right)\right]}^{T}={\left[2.5,-2.5,0.5\right]}^{T}.$$

The reference trajectory's desired velocities are *v*_*d*_ = 0.5 and *w*_*d*_ = 0.04. Under the input constraints, ℏ = 1.5, meaning the constraint range is [−1.5, 1.5]. The unreliable communication γ is defined as


$$\gamma \left(t\right)=\sigma \left[\begin{array}{c} \sin\left(\sigma \right)x_{e}\\ \cos\left(\sigma \right)y_{e}\\ \sin\left(\sigma \right)x_{e}y_{e} \end{array}\right],$$

where σ is a random variable uniformly distributed in σ ∈ [−0.1, 0.1]. For the performance evaluation, we define the cost function using the weighting matrices


$$Q=\left[\begin{array}{ccc} 10 & 0 & 0\\ 0 & 10 & 0\\ 0 & 0 & 10 \end{array}\right],\;R=\left[\begin{array}{cc} 1.5 & 0\\ 0 & 0.5 \end{array}\right].$$

The activation function vector of CNN is $\varphi \left(z\right)=[z_{1}^{4},z_{2}^{4},z_{3}^{4},$ $z_{1}^{2}z_{2}^{2},z_{2}^{2}z_{3}^{2},z_{1}^{2}z_{3}^{2},z_{1}^{2}z_{2},z_{2}^{2}z_{3},z_{1}z_{2}^{2},z_{3}^{3},$ sin(*z*_1_), sin(*z*_2_), sin(*z*_3_), $\cos\left(z_{1}\right),\cos\left(z_{2}\right),\cos\left(z_{3}\right){]}^{T}$. ρ_1_ = 1.1, ρ_2_ = 1.03, ϱ_1_ = 3.2, ϱ_2_ = 1.08, $\delta_{1}=0.3\times 10^{-5}$, $\delta_{2}=0.4\times 10^{-2}$, *A* = 1, and *B* = 1.

**Figure 3:**
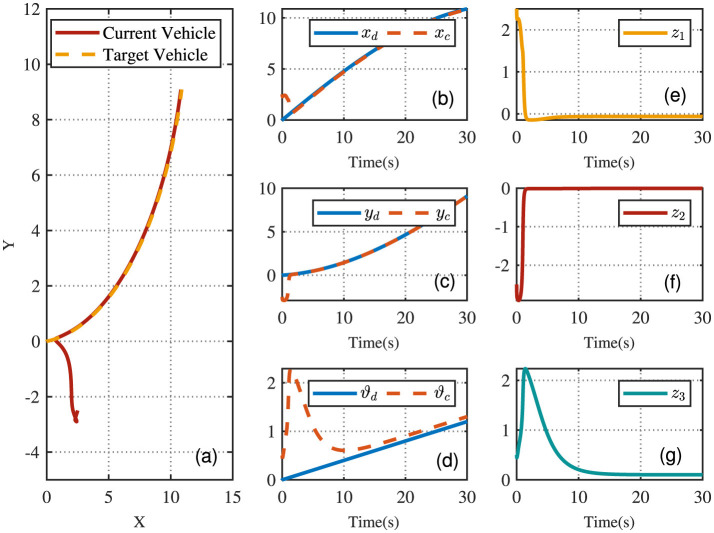
AVR driving trajectories. **(A)** The X-Y plot of tracking trajectories. **(B–D)** Tracking trajectories. **(E–G)** Tracking errors.

Using the proposed method, Figure 3A illustrates the two-dimensional trajectory of the AVR. The vehicle quickly adjusts its direction and begins tracking the reference trajectory with good accuracy. After the initial phase, the vehicle follows the desired trajectory smoothly and closely. Figures 3B–G depict the tracking performance and error, demonstrating that the position error gradually reduces to zero, while the directional error also diminishes to zero, effectively ensuring precise position tracking throughout the process.

**Figure 4:**
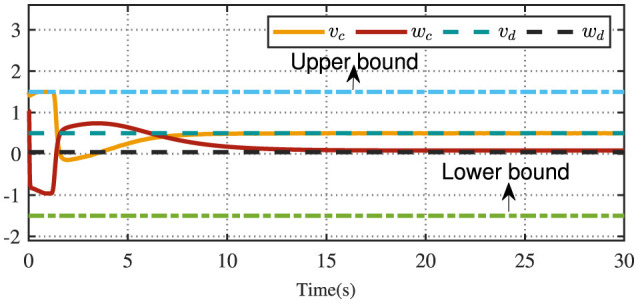
The constrained control input.

**Figure 5:**
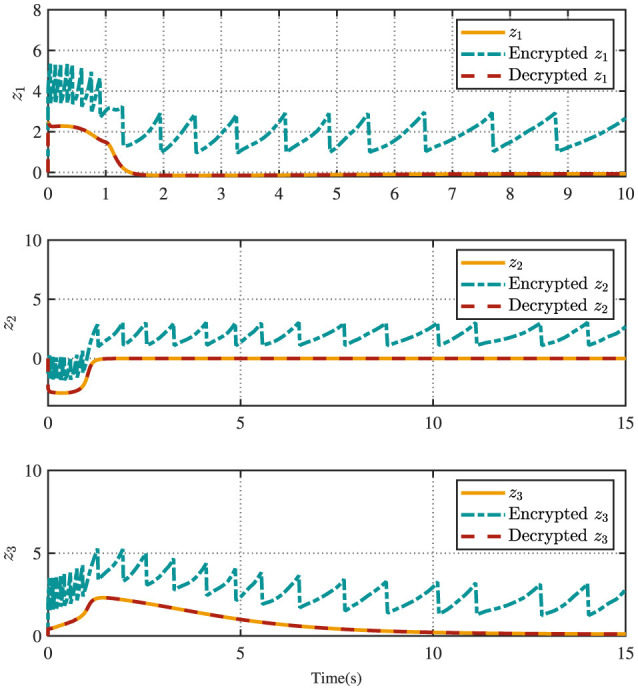
Encrypted error and decrypted error.

**Figure 6:**
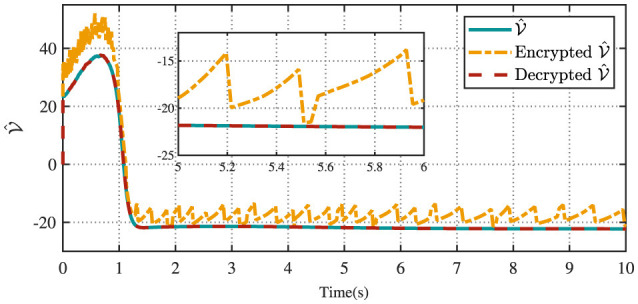
Encrypted value function and decrypted value function.

Figure 4 displays the evolution of the designed controller during the vehicle's tracking process. The dashed lines indicate the upper and lower bounds of the input constraints, which are set to [−1.5, 1.5]. The privacy-preserving characteristics of the proposed scheme are illustrated in Figure 5. It is evident that masking the vehicle-side output *z* effectively safeguards its privacy from potential attackers. Meanwhile, as shown in Figure 6, masking on the cloud side further prevents the leakage of critical information related to the designed control strategy. Therefore, these results ensure robust privacy protection during data transmission.

## 6 Conclusion

This study develops an encrypted guaranteed-cost tracking control scheme to address the challenges of information security and computational efficiency in AVR systems using the adaptive dynamic programming technique. By leveraging ADP and integrating encryption mechanisms between the vehicle and the cloud, the proposed method ensures stable tracking performance under unreliable communication. The input constraints are successfully managed using a nonlinear value function, while the CNN facilitates an efficient solution to the HJB equation. Simulation results from a case study confirm the stability and effectiveness of the designed algorithm, demonstrating its potential for real-world applications in AVR networks. Future work will focus on ensuring the security of cloud-based computations by processing encrypted data, further enhancing the safety and reliability of cloud operations in vehicular network systems.

## Data Availability

The original contributions presented in the study are included in the article/supplementary material, further inquiries can be directed to the corresponding author.
